# Test-retest reproducibility of [^11^C]-l-deprenyl-D_2_ binding to MAO-B in the human brain

**DOI:** 10.1186/s13550-017-0301-4

**Published:** 2017-06-20

**Authors:** Ryosuke Arakawa, Per Stenkrona, Akihiro Takano, Sangram Nag, Rafael S. Maior, Christer Halldin

**Affiliations:** 1 Department of Clinical Neuroscience, Center for Psychiatry Research, Karolinska Institutet and Stockholm County Council, Stockholm, Sweden; 2 Primate Center and Laboratory of Neurosciences and Behavior, Department of Physiological Sciences, Institute of Biology, University of Brasilia, Brasilia, Brazil

**Keywords:** Age effect, [^11^C]-l-deprenyl-D_2_, Monoamine oxidase B, Partial volume effect correction, Positron emission tomography, Test-retest variability

## Abstract

**Background:**

[^11^C]-l-deprenyl-D_2_ is a positron emission tomography (PET) radioligand for measurement of the monoamine oxidase B (MAO-B) activity in vivo brain. The estimation of the test-retest reproducibility is important for accurate interpretation of PET studies.

**Results:**

We performed two [^11^C]-l-deprenyl-D_2_ scans for six healthy subjects and evaluated the test-retest variability of this radioligand. MAO-B binding was quantified by two tissue compartment model (2TCM) with three rate constants (*K*_1_, *k*_2_, *k*_3_) using metabolite-corrected plasma radioactivity. The *λk*_3_ defined as (*K*_1_/*k*_2_) × *k*_3_ was also calculated. The correlation between MAO-B binding and age, and the effect of partial volume effect correction (PVEc) for the reproducibility were also estimated. %difference of *k*_3_ was 2.6% (medial frontal cortex) to 10.3% (hippocampus), and that of *λk*_3_ was 5.0% (thalamus) to 9.2% (cerebellum). Mean %difference of all regions were 5.3 and 7.0% in *k*_3_ and *λk*_3_, respectively. All regions showed below 10% variabilities except the hippocampus in *k*_3_ (10.3%). Intraclass correlation coefficient (ICC) of *k*_3_ was 0.78 (hippocampus) to 0.98 (medial frontal cortex), and that of *λk*_3_ was 0.78 (hippocampus) to 0.95 (thalamus). Mean ICC were 0.94 and 0.89 in *k*_3_ and *λk*_3_, respectively. The highest positive correlation with age was observed in the hippocampus, as *r* = 0.75 in *k*_3_ and 0.76 in *λk*_3_. After PVEc, mean %difference were 5.6 and 7.2% in *k*_3_ and *λk*_3_, respectively. Mean ICC were 0.92 and 0.90 for *k*_3_ and *λk*_3_, respectively. These values were almost the same as those before PVEc.

**Conclusions:**

The present results indicate that *k*_3_ and *λk*_3_ of [^11^C]-l-deprenyl-D_2_ are reliable parameters for test-retest reproducibility with healthy subjects both before and after PVEc. The studies with patients of larger sample size are required for further clinical applications.

## Background

Monoamine oxidase (MAO) is an enzyme of deamination of monoamine and exists in two isoforms, MAO-A and MAO-B. MAO-B is considered as a major enzyme in dopamine metabolism in the human brain, and MAO-B inhibitors are used for the treatment of Parkinson’s disease to increase the dopamine level [1]. For Alzheimer’s disease (AD), several studies reported the upregulation of MAO-B expression related to the neuroinflammatory process in the glia cells [2–6]. So, inhibition of MAO-B is expected to have beneficial effects in AD by reducing neurodegenerative processes.

[^11^C]-l-deprenyl-D_2_ is a positron emission tomography (PET) radioligand for measurement of the MAO-B activity in the brain [7]. This radioligand was used for many PET studies to evaluate MAO-B inhibitors’ occupancy [8, 9], pathology of AD [10–12], amyotrophic lateral sclerosis (ALS) [13], traumatic brain injury [14], and epilepsy [15–17]. In addition to the clinical studies, [^11^C]-l-deprenyl-D_2_ showed good result reproducibility in a test-retest protocol [18].

Recently, we reported the brain occupancy of MAO-B using [^11^C]-l-deprenyl-D_2_ after administration of a MAO-B inhibitor, sembragiline, in controls and patients with AD [9]. Although the good reproducibility of [^11^C]-l-deprenyl-D_2_ has already been reported, confirmation in test-retest variability studies by using identical protocol settings, including radioligand production and PET machine, is helpful for accurate interpretation of occupancy. Additionally, age effect of [^11^C]-l-deprenyl-D_2_ should be estimated since an increase of MAO-B expression has been reported in elderly subjects by postmortem [19] and in vivo PET [20] studies. The partial volume effect (PVE) should be also considered in the PET studies for aged subjects or AD patients with brain atrophy [21, 22].

In this study, we focused on three issues. First, we evaluated the test-retest variability of [^11^C]-l-deprenyl-D_2_ in the healthy subjects who were scanned twice. Second, the correlation between MAO-B activity and age was estimated. Finally, the effect of partial volume effect correction (PVEc) for the reproducibility was also estimated.

## Methods

### Subjects

Six healthy volunteers (age range, 21–62 years; mean ± SD, 39.0 ± 17.0; four males, two females) were enrolled in the study. None had a history of present or past psychiatric, neurological, or somatic disorders, or alcohol or drug-related problems. They took no medication at the time of the study. After thorough explanation of the study, written informed consent was obtained from all participants. The study was approved by the Regional Ethical Review Board in Stockholm, Sweden, and the Radiation Safety Committee at the Karolinska University Hospital Solna in Stockholm, Sweden.

### PET procedures

[^11^C]-l-deprenyl-D_2_ was prepared by *N*-methylation of the desmethyl l-deprenyl-D_2_ precursor using [^11^C]methyl triflate as previously reported [9]. PET scans were carried out with the ECAT Exact HR 47 (Siemens/CTI, Knoxville, TN, USA), with an in-plane and an axial resolution of 3.6 and 4.0 mm at full width at half maximum (FWHM), respectively. A head fixation system with an individual plaster helmet was used. A 5-min transmission scan was performed using three rotating ^68^Ge rod sources. Dynamic PET scan was performed for 63 min (10 s × 9, 15 s × 2, 20 s × 3, 30 s × 4, 1 min × 4, 3 min × 4, 6 min × 7) after intravenous bolus injection of [^11^C]-l-deprenyl-D_2_. Injected radioactivity was 299–520 MBq (419 ± 68 MBq), which were adjusted by body weight. Specific radioactivity was 104–341 GBq/μmol (179 ± 80 GBq/μmol) at the time of injection. Injected mass was 0.24–0.77 μg (0.50 ± 0.18 μg). Two PET scans were performed for each subject in the morning and in the afternoon on the same day.

### Arterial blood sampling

Arterial blood was collected continuously for 10 min using an automated blood sampling system at a speed of 5 mL/min. Arterial blood was also drawn manually 1, 2, 4, 6, 8, 10, 15, 20, 30, 45, and 60 min after the injection for measurement of radioactivity in whole blood and plasma. The parent fraction of the radioligand was determined using a reversed-phase high-performance liquid chromatography (HPLC) method at 4, 10, 20, 30, 45, and 60 min. Plasma input function was defined as radioactivity of plasma multiplied by the percentage of unchanged radioligand.

### Data analysis

PET images were reconstructed using the standard filtered back projection with a 2-mm Hanning filter, a zoom factor of 2.17, and a 128 × 128 matrix, and were corrected for attenuation and scatter. T1-weighted magnetic resonance imaging (MRI) was performed using a 1.5-T Siemens MAGNETOM Avanto for definition of anatomical brain regions and PVEc. The MR image was segmented into grey matter (GM), white matter (WM), and cerebrospinal fluid (CSF) segments, using the SPM5 segmentation algorithm in MATLAB (Wellcome Trust Centre for Neuroimaging, London, UK; The MathWorks, Inc., Natick, MA, USA). Regions of interest (ROI) were defined as the cerebellum, hippocampus, lateral frontal cortex, lateral occipital cortex, lateral parietal cortex, lateral temporal cortex, medial frontal cortex, putamen, and thalamus using the Anatomical Automatic Labeling (AAL) template [23]. GM masking was applied to the AAL template. All MRIs and ROIs were co-registered to summated PET images with the mutual information algorithm using SPM5. Regional radioactivity was calculated for each frame, corrected for decay, and plotted vs. time.

MAO-B binding of [^11^C]-l-deprenyl-D_2_ was quantified by two tissue compartment model (2TCM) with three rate constants (*K*_1_, *k*_2_, *k*_3_) using metabolite-corrected plasma radioactivity as the input function. *k*_4_ was set as 0 according to the assumption of irreversible binding of [^11^C]-l-deprenyl-D_2_ [7, 18]. Blood volume was fixed as 5%. The *λk*_3_ defined as (*K*_1_/*k*_2_) × *k*_3_ was also calculated. These analyses were performed using the PMOD 3.4 software package (PMOD Group, Zurich, Switzerland).

### PVEc

All PET data were corrected for PVE according to the method reported by Müller-Gärtner et al. [21]. In short, this PVEc corrects for spillover between GM and WM, and between GM and CSF using the segmented MR images. By assuming that the radioligand binding in WM is perfectly homogenous, every WM-voxel were replaced by an average value derived from an isolated proportion of WM at the centrum semiovale [22].

### Statistics

The test-retest variability of MAO-B binding as *k*_3_ and *λk*_3_ was expressed by the absolute difference between the first and second PET scans (PET1 and PET2) relative to the mean of the two values according to the following equation [24, 25]:

$$ \%\mathrm{difference}=\left|\mathrm{PET}1-\mathrm{PET}2\right|/\left(\left(\mathrm{PET}1+\mathrm{PET}2\right)/2\right)\times 100 $$

PET1 and PET2 are the parameters (*k*_3_ or *λk*_3_) of first and second PET scans, respectively.

Intraclass correlation coefficient (ICC) was also calculated by:

$$ \mathrm{I}\mathrm{C}\mathrm{C}=\left(\mathrm{MSBS}-\mathrm{MSWS}\right)/\left(\mathrm{MSBS}+\mathrm{MSWS}\right) $$

MSBS is the mean square of between subject, and MSWS is the mean square of within subject.

The correlation between MAO-B binding as *k*_3_ or *λk*_3_ and age was estimated using the Pearson correlation coefficient.

The effect of PVEc was estimates by the %change of before and after PVEc of all parameters, *K*_1_, *k*_2_, *k*_3_, and *λk*_3_:

$$ \%\mathrm{change}=\left(\mathrm{after}\ \mathrm{PVEc}-\mathrm{before}\ \mathrm{PVEc}\right)/\mathrm{before}\ \mathrm{PVEc}\times 100 $$

In both analysis of age effect and PVEc, average values of PET1 and PET2 were used. Finally, the test-retest variability of after PVEc was estimated as same way as before PVEc.

These statistical analyses were performed using R 3.3.2 (The R Foundation, Vienna, Austria).

## Results

### Test-retest variability

Mean and SD values of six subjects in PET1 and PET2, mean and range of %differences, and ICC in all regions are shown in Table 1. %difference of *k*_3_ was 2.6% (medial frontal cortex) to 10.3% (hippocampus), and that of *λk*_3_ was 5.0% (thalamus) to 9.2% (cerebellum). Mean %difference of nine regions were 5.3% and 7.0% in *k*_3_ and *λk*_3_, respectively. All regions showed variability below 10%, except the hippocampus in *k*_3_ (10.3%). ICC of *k*_3_ was 0.78 (hippocampus) to 0.98 (medial frontal cortex), and that of *λk*_3_ was 0.78 (hippocampus) to 0.95 (thalamus). Mean ICC of nine regions were 0.94 and 0.89 in *k*_3_ and *λk*_3_, respectively.

**Table 1:** Mean and SD values in PET1 and PET2, mean and range of %differences, and ICC in all regions of *k*_3_ and *λk*_3_

	*k*_3_							*λk*_3_						
	PET1		PET2		%dif			PET1		PET2		%dif		
Region	Mean	SD	Mean	SD	Mean	Range	ICC	Mean	SD	Mean	SD	Mean	Range	ICC
Cerebellum	0.051	0.011	0.051	0.012	5.1	0.7–11.1	0.96	0.143	0.030	0.140	0.031	9.2	5.9–15.4	0.90
Hippocampus	0.103	0.023	0.108	0.017	10.3	0.2–20.6	0.78	0.288	0.028	0.285	0.047	6.4	1.0–17.6	0.78
Lateral frontal cortex	0.067	0.014	0.067	0.013	3.8	0.4–7.2	0.97	0.185	0.023	0.184	0.033	6.0	2.4–14.2	0.91
Lateral occipital cortex	0.060	0.013	0.061	0.013	6.4	1.0–15.1	0.92	0.175	0.024	0.170	0.030	8.0	3.0–15.0	0.86
Lateral parietal cortex	0.063	0.012	0.064	0.012	3.7	1.4–7.7	0.97	0.180	0.027	0.182	0.035	7.2	2.5–12.1	0.91
Lateral temporal cortex	0.065	0.015	0.063	0.015	5.9	0.1–13.7	0.95	0.201	0.029	0.199	0.041	8.3	2.2–20.0	0.86
Medial frontal cortex	0.068	0.012	0.067	0.011	2.6	1.1–4.6	0.98	0.175	0.019	0.175	0.026	5.7	2.8–10.1	0.89
Putamen	0.087	0.019	0.085	0.016	5.4	3.2–7.6	0.96	0.323	0.051	0.331	0.072	7.4	3.0–10.5	0.91
Thalamus	0.083	0.017	0.082	0.017	4.2	0.1–9.4	0.97	0.306	0.047	0.308	0.059	5.0	1.5–8.6	0.95

### Age effect

**Fig. 1:**
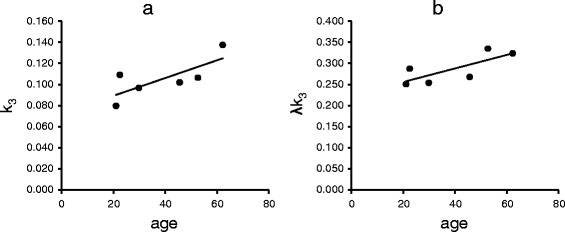
The relationship between age and **a** *k*_3_ or **b** *λk*_3_ of six subjects

The Pearson correlation coefficient and *p* value between *k*_3_ or *λk*_3_ and age in all regions are shown in Table 2. The highest positive correlation was observed in the hippocampus (*r* = 0.75 in *k*_3_ and 0.76 in *λk*_3_; Fig. 1a, b). Regardless, both results were not statistically significant (*p* = 0.08 and *p* = 0.08, respectively).

**Table 2:** The Pearson correlation coefficient and *p* value between age and parameters in all regions of *k*_3_ and *λk*_3_

	*k*_3_		*λk*_3_	
Region	*r*	*p*	*r*	*p*
Cerebellum	0.58	0.23	0.56	0.25
Hippocampus	0.75	0.08	0.76	0.08
Lateral frontal cortex	0.65	0.17	0.73	0.10
Lateral occipital cortex	0.66	0.16	0.59	0.22
Lateral parietal cortex	0.70	0.13	0.64	0.18
Lateral temporal cortex	0.69	0.13	0.72	0.10
Medial frontal cortex	0.58	0.22	0.66	0.15
Putamen	0.67	0.15	0.75	0.09
Thalamus	0.69	0.13	0.63	0.18

### PVEc

**Fig. 2:**
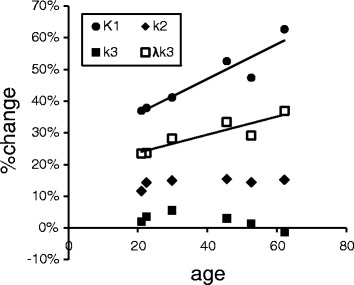
The relationship between age and %change of parameters (*K*_1_, *k*_2_, *k*_3_, and *λk*_3_) after PVEc of six subjects

After PVEc, *K*_1_ values increased as 36.9 to 62.7% according to the age (Fig. 2). %change of *λk*_3_ was 23.5 to 37.0% and also correlated with age. Compared to *K*_1_ and *λk*_3_, *k*_2_ and *k*_3_ showed only small changes after PVEc (11.6 to 15.4% and −1.3 to 5.5%, respectively).

Mean and SD values of six subjects in PET1 and PET2, mean and range of %differences, and ICC after PVEc in all regions are shown in Table 3. Mean %difference of nine regions were 5.6 and 7.2% in *k*_3_ and *λk*_3_, respectively. Mean ICC of nine regions were 0.92 and 0.90 in *k*_3_ and *λk*_3_, respectively. These values were almost the same as those before PVEc.

**Table 3:** Mean and SD values in PET1 and PET2, mean and range of %differences, and ICC in all regions of *k*_3_ and *λk*_3_ after PVEc

	*k*_3_							*λk*_3_						
	PET1		PET2		%dif			PET1		PET2		%dif		
Region	Mean	SD	Mean	SD	Mean	Range	ICC	Mean	SD	Mean	SD	Mean	Range	ICC
Cerebellum	0.050	0.011	0.051	0.012	5.6	1.1–11.2	0.95	0.174	0.039	0.170	0.040	9.8	5.6–16.1	0.91
Hippocampus	0.116	0.024	0.122	0.019	10.6	0.3–23.2	0.70	0.345	0.028	0.340	0.055	7.4	1.4–17.7	0.73
Lateral frontal cortex	0.065	0.012	0.065	0.012	4.0	1.5–7.1	0.97	0.272	0.050	0.271	0.063	6.2	2.0–15.0	0.95
Lateral occipital cortex	0.059	0.010	0.059	0.012	7.2	1.4–16.7	0.86	0.243	0.039	0.233	0.047	9.1	1.3–19.8	0.84
Lateral parietal cortex	0.062	0.011	0.064	0.011	4.0	1.3–7.2	0.96	0.265	0.049	0.267	0.059	7.7	2.9–12.3	0.93
Lateral temporal cortex	0.065	0.014	0.063	0.014	6.0	0.4–14.4	0.95	0.264	0.046	0.260	0.061	9.0	2.1–22.8	0.88
Medial frontal cortex	0.065	0.010	0.065	0.010	2.2	0.5–4.2	0.98	0.265	0.041	0.267	0.052	5.1	0.0–9.1	0.94
Putamen	0.089	0.018	0.086	0.017	4.8	1.1–7.6	0.96	0.329	0.053	0.337	0.076	7.0	0.4–12.0	0.91
Thalamus	0.095	0.020	0.096	0.018	6.3	1.7–10.5	0.92	0.326	0.061	0.325	0.072	3.9	0.0–8.5	0.96

## Discussion

In this study, the test-retest variability of [^11^C]-l-deprenyl-D_2_ was estimated using healthy subjects. Mean of absolute %difference in nine regions of *λk*_3_ was 7.0%, indicating good reproducibility. The test-retest variability of *λk*_3_ using %difference (not absolute difference) by Logan et al. was previously reported as −2.84 ± 7.07% [18]. The same calculation of our data yielded −1.1 ± 8.3%, indicating similar variability. ICC values were high in all regions, except in the hippocampus which was relatively low (0.78). It also indicated good reproducibility of [^11^C]-l-deprenyl-D_2_. Our research group recently reported the brain occupancy of MAO-B using [^11^C]-l-deprenyl-D_2_ after administration of a MAO-B inhibitor—sembragiline [9]. The study reported that oral administrations of 1–5 mg sembragiline induced near-maximal inhibition of brain MAO-B, and MAO-B occupancy was correlated with the plasma concentration of sembragiline in patients with AD. The good test-retest reproducibility of the present study using same procedure of [^11^C]-l-deprenyl-D_2_ preparation and same PET camera corroborates the findings of our previous study.

Although *λk*_3_ is generally considered to be a more stable outcome measure than *k*_3_ [7, 18], *k*_3_ showed similar %difference as *λk*_3_ (5.3 vs 7.0%) in the present study. *k*_3_ values also showed better ICC than those of *λk*_3_ (mean value, 0.94 vs 0.89). These findings indicate that *k*_3_ may be independently used for further studies such as comparison between patients and healthy controls or target occupancy of MAO-B inhibitors. Obvious regional differences of reproducibility were only observed in the hippocampus. The reason of low reproducibility in the hippocampus is not clear, but one possibility is that its high *k*_*3*_ value (the highest among all regions) may have induced a large variability.

In this study, two PET measurements were performed in 1 day, in the morning and afternoon. Several studies suggested that dopamine system was affected by the circadian rhythm [26–28]. Especially, one rodent study reported that MAO-A expression was regulated by the clock-component gene [29]. However, our result did not show the relation between the MAO-B binding and the time of PET measurements.

A moderate age effect on *λk*_3_ values was observed in all regions. The degree of increase (6.3% per decade) was similar as the previously reported value by Fowler et al., at 7.1% per decade [20]. One postmortem study also reported increasing of MAO-B protein in the human brain according to age [19]. Our PET study confirmed both findings of postmortem and in vivo human brain studies. Among all regions, the hippocampus showed the highest correlation with age effect although it did not reach statistical significance and the reproducibility was relatively low as discussed above. Since the hippocampus is thought to be especially impacted in AD [30, 31], the present findings suggest the increase of MAO-B activity may have an important role about the pathology of AD.

PVE should be considered in PET studies when brain atrophy is observed in aged subjects or patients with disorders such as AD. Additionally, PVE is larger using a low resolution PET camera than a high resolution one. This study included elderly subjects and was conducted using relatively low resolution PET camera system compared to recent PET systems like the high resolution research tomograph (HRRT) with 1.5 mm FWHM [32]. In the present study, %change of *K*_1_ after PVEc was correlated with age. It indicates that the brain of aged subjects was more affected by the PVE compared to younger subject due to the brain atrophy. Meanwhile, *k*_2_ and *k*_3_ did not show any clear change after PVEc. *λk*_3_ was also moderately affected by PVEc and correlated with age since it is partially determined by *K*_1_/*k*_2_. Nevertheless, although the value itself of *λk*_3_ was increased in aged subjects, test-retest variability of *λk*_3_ did not change after PVEc. This indicates that PVEc can reliably keep reproducibility even in the case of elderly subjects with brain atrophy.

There are several imitations in this study. Although only six subjects were included and it is relatively small sample size, the number were similar as previous test-retest studies about [^11^C]-l-deprenyl-D_2_ by Logan et al. (*n* = 5) [18] and other PET radioligands (*n* = 6–8) [25, 33]. However, the studies about age effect included larger number generally [20, 34, 35]. So, the present result about the age effect might be preliminary. Additionally, only healthy control subjects were included in this study. It is difficult to extrapolate our findings about PVEc to AD patients because the elderly ones showed slight atrophy but not pathological. To more accurately estimate the effects of PVEc, further studies with AD patients and aged control subjects who showed strong atrophy will be needed.

## Conclusions

The present results indicate that *k*_3_ and *λk*_3_ of [^11^C]-l-deprenyl-D_2_ are reliable parameters for test-retest reproducibility with healthy subjects both before and after PVEc. As the number of subjects was relatively small and only healthy subjects were included, the studies with patients of larger sample size are required for further clinical applications.

## Abbreviations

2TCM: Two tissue compartment model
AAL: Anatomical Automatic Labeling
AD: Alzheimer’s disease
ALS: Amyotrophic lateral sclerosis
CSF: Cerebrospinal fluid
FWHM: Full width at half maximum
GM: Grey matter
HRRT: High resolution research tomograph
ICC: Intraclass correlation coefficient
MAO: Monoamine oxidase
MRI: Magnetic resonance image
MSBS: Mean square of between subject
MSWS: Mean square of within subject
PET: Positron emission tomography
PVE: Partial volume effect
PVEc: Partial volume effect correction
ROI: Regions of interest
WM: White matter
